# Multicomponent family support intervention in intensive care units: statistical analysis plan for the cluster-randomized controlled FICUS trial

**DOI:** 10.1186/s13063-024-08351-y

**Published:** 2024-08-28

**Authors:** Stefanie von Felten, Miodrag Filipovic, Marie-Madlen Jeitziner, Lotte Verweij, Marco Riguzzi, Rahel Naef

**Affiliations:** 1 Department of Biostatistics at Epidemiology, Biostatistics and Prevention Institute, University of Zurich, Hirschengraben 84, Zurich, CH-8001 Switzerland; 2 Division of Perioperative Intensive Care Medicine, Kantonsspital St. Gallen, Rorschacher Strasse 95, St. Gallen, CH-9007 Switzerland; 3 Department of Intensive Care Medicine, Inselspital, University Hospital Bern, Freiburgstrasse 10, Bern, CH-3010 Switzerland; 4 Institute for Implementation Science in Health Care, Faculty of Medicine, University of Zurich, Universitätstrasse 84, Zurich, CH-8006 Switzerland; 5 Centre of Clinical Nursing Science, University Hospital Zurich, Rämistrasse 100, Zurich, CH-8091 Switzerland

**Keywords:** Statistical analysis plan, Cluster-randomized trial, Critical care, Complex intervention, Family care

## Abstract

The FICUS trial is a cluster-randomized superiority trial to determine the effectiveness of a nurse-led, interprofessional family support intervention (FSI) on the quality of care, family management and individual mental health of family members of critically ill patients, compared to usual care. This paper describes the statistical analysis plan of the FICUS trial. The primary outcome is quality of family care, assessed by the Family Satisfaction in ICU Questionnaire (FS-ICU-24R) at patient discharge from the ICU. Several secondary outcomes are additionally assessed 3, 6, and 12 months thereafter. Sixteen clusters (ICUs) were randomly assigned 1:1 to FSI or usual care using minimization (8 per treatment). The target sample size is 56 patients per cluster (896 in total). Recruitment has been completed in January 2024. The follow-up of the last participant will be completed in early 2025. The primary and secondary outcomes will be analyzed by linear mixed-effects models (LMM). The main model for the primary outcome will include a random intercept per cluster with treatment (FSI vs. usual care) as the only explanatory variable due to the relatively small number of clusters. In addition, covariate-adjusted analyses will be conducted, including two cluster-level characteristics used in the minimization as well as participant-level characteristics. Moreover, a number of subgroup analyses by cluster- and participant-level characteristics are pre-specified.

**Trial registration** ClinicalTrials.gov NCT05280691. Registered on February 20, 2022.

## Introduction

### Background and rationale for trial

The admission of a patient to an intensive care unit (ICU) creates enormous uncertainty and stress among family members [1–3]. Critical illness is life-altering and often life-threatening, which exposes families to extraordinary challenges for which they are often unprepared [4, 5]. A close other’s critical illness is associated with emotional distress and negative mental health outcomes for families [6–9]. When affected by critical illness, families have specific needs, such as (1) being with their critically ill close other to provide meaningful support, (2) developing trustful partnerships with ICU staff to receive ongoing information on the patient’s condition and prognosis and ensure communication among involved parties, and (3) receiving guidance and support in dealing with the enormous challenges they are facing during and after the critical illness phase [4, 10–15]. Structured support for families is therefore called for [16–19].

Guidelines for family-centered care in the ICU recommend proactive engagement, communication and support to families, and the use of consultations and specific family navigator roles [20, 21], which has only been partially implemented to date [19, 20]. Moreover, there is insufficient empirical evidence on the clinical effectiveness of specific family support interventions that combine these different family care practices into a program of family care. Therefore, the Family support intervention in Intensive Care UnitS (FICUS) trial investigates the clinical effectiveness of a multi-component, nurse-led, interprofessional family support intervention (FSI) in addition to usual care [22].

### Objectives

The primary objective is to show that the FSI, in addition to usual care, improves the quality of family care in ICU, assessed as family members’ satisfaction with care at patient ICU discharge, compared to usual care alone. The secondary objective is to test the effectiveness of the FSI on further indicators of quality of care assessed by family members at ICU discharge, as well as on family management of critical illness and family members’ post-ICU mental health, assessed at patient ICU admission (baseline), patient ICU discharge, and 3, 6, and 12 months following ICU discharge.

## Study methods

### Trial design

The FICUS trial is a parallel, cluster-randomized, controlled, multicenter, superiority trial with an equal number of clusters per study arm (8 ICUs each). The effectiveness-implementation hybrid type 1 design [23] examines, in addition to clinical effectiveness, the implementation of the FSI in the dynamic and complex “real-world” context of ICU care. However, this statistical analysis plan is focused on analyses of effectiveness. The intervention (FSI) consists of multiple components of family engagement, support, and communication provided by designated family nurses and members of the interprofessional team along the patient pathway, including follow-up care. The FSI is manual-based and includes (1) early engagement and liaison with families over time, (2) psycho-educational and relationship-focused family interventions in the form of therapeutic conversations, and (3) structured, interprofessional communication, and shared decision-making with families [22]. Families in the control arm receive usual care. While several members of a family receive the intervention, only one family member per patient is included in the study.

After the collection of baseline data (at patient admission to ICU, T0), outcomes are assessed at patient discharge from ICU (T1, primary outcome) as well as 3, 6, and 12 months thereafter (T2, T3, and T4, respectively), using established psychometric family-reported outcome measures. In addition, patient-related data are retrieved from clinical records at T0 and T1.

### Randomization

Clusters (ICUs) were assigned 1:1 to the intervention or the control arm using minimization. The variables used in the minimization procedure are the certification of the ICU (2 groups) and hospital (one hospital included 2 ICUs and one hospital included 4 ICUs). We originally planned to define degree of specialization as specialized vs. general ICU [22] but finally used the ICU certification according to the registry of the certified training centers of the Swiss Institute for Continuing Medical Education (https://www.siwf-register.ch). The classification depends on the size (total number of ICU treatment days/mechanical ventilation hours), case mix, hospital infrastructure, and possibility for scientific activity and approximately divides hospitals into major teaching hospitals (A/Au, larger cantonal and university hospitals) vs. other teaching hospitals (B, smaller cantonal and regional hospitals). The minimization was prepared by SvF using the R package Minirand [24, 25]. A random seed was used to make the minimization procedure reproducible, which was generated by MR rolling a 20-sided die three times resulting in a six-digit number. To avoid the minimization process to be fully deterministic, we used a random component of 10%. Due to the relatively small number of clusters, we determined a priori that the whole randomization process including setting the seed would be repeated if (and only if) ICUs from the same hospital were not assigned 1:1 or the clusters overall were not assigned 1:1 (8 each to the intervention and control arm). SvF and MR performed the minimization on February 24, 2022.

### Sample size

The sample size calculation is described in more detail in the published study protocol [22]. Using the R package clusterPower [26], we determined that with an average cluster size of 50 evaluable participants, a coefficient of variation in cluster size of 0.2, a difference in the primary outcome (FS-ICU-24 total score) between groups of 5.5, a within-group standard deviation of 16.3, and an ICC of 0.03, 8 clusters need to be assigned to each study arm (16 in total) to achieve a power of 80% at a significance level of 5%. To account for a drop-out rate of 10% of participants within clusters (but no drop-out of entire clusters), an average number of 56 participants per cluster should be recruited.

### Framework

The FICUS trial aims to demonstrate the superiority of the FSI in comparison to usual care in the ICUs for the primary outcome (assessed at T1 only). Secondary outcomes will also be compared using a superiority framework.

### Statistical interim analyses and stopping guidance

No interim analyses are planned.

### Timing of final analysis

The effectiveness of the FSI on the primary outcome and secondary outcomes only assessed once at patient discharge from the ICU (T1) will be analyzed after all study centers have completed recruitment (last participant-in) and all participants have completed the first follow-up data collection (T1). All other analyses of FSI effectiveness will be performed after completion of the trial, i.e., after all participants have reached 1 year of follow-up after patient discharge from ICU (T4).

### Timing of outcome assessments

The primary outcome, family satisfaction with ICU care (FS-ICU-24R), as well as the secondary outcomes of quality of communication (QQPPI-14) and nurse support (ICE-FPSQ-14) are assessed at patient discharge from ICU only (T1). All other secondary outcomes concern family and mental health measures and are assessed at T1 as well as 3, 6, and 12 months thereafter (T2–T4). For the secondary outcomes assessed at multiple time points, an assessment at baseline is also performed within 4 days after admission to ICU (T0). The originally planned time windows for the outcome assessments were a maximum of 1 day before and up to 14 days after ICU discharge (T1), and 90, 180, and 365 days after T1 ± 14 days for T2–T4, respectively. However, to reduce the amount of missing data due to later return of questionnaires, these time windows were adapted as follows:

- T1—discharge from ICU (−1 day/$+$90 days, completed before T2 for quality of family care indicators; −1 day/$+$4 weeks for family and mental health indicators)
- T2—3 months following T1 (90 days; −14 days/$+$4 weeks)
- T3—6 months following T1 (180 days; −14 days/$+$6 weeks)
- T4—12 months following T1 (365 days; −14 days/$+$6 weeks)

The relatively large time window of $+$90 days for the quality of family care indicators was extended compared to the time window specified in the published study protocol [22] because of high burden in family members, which is likely to lead to a return of questionnaires later than the originally planned 2 weeks, but will hardly lead to a recall bias. It is important to note that study endpoints in the family and mental health indicators, which are measured at several time points and are likely to fluctuate over time, have a narrower time-window at T1 and also at T2–T4.

## Statistical principles

### Level of statistical significance

The significance level for the primary outcome and the secondary outcomes will be set at 0.05 (two-sided).

### Adjustment for multiplicity

No adjustments for multiplicity are planned. Tests for the primary outcome will be considered confirmatory, whereas tests for the secondary outcomes and subgroup analyses will be considered exploratory. All planned analyses will be reported. All *p*-values will be reported with two significant digits if $\ge 0.0001$, and as $<0.0001$ otherwise.

### Confidence intervals to be reported

We will report 95% confidence intervals for all point estimates.

### Adherence and protocol deviations

The intervention is standardized in terms of components, intervention content, and a minimum dose along the clinical patient pathway (i.e., at least five intervention contacts/doses, representing all three intervention components of “engaging & liaising,” “supporting,” and “communicating”; see Fig. 3 in the published study protocol [22]). Interventionists tailor the frequency of intervention contacts and the dose of each intervention component according to a patient’s course of illness and/or a family’s preferences and needs, thereby potentially increasing dose and frequency.

Adherence is defined as fidelity to the manual-based study intervention (=fidelity consistency). Consistent intervention fidelity has been reached when the participant has received the protocolized minimum dose of the five intervention contacts, representing all three intervention components, within the specified time frame along the patient pathway [22]. Adherence is not defined under usual care (control). We will report the number and percentage of participants who did or did not receive the minimum dose of the FSI in the intervention arm.

Relevant deviations from the study protocol were defined as follows:

- If an enrolled participant is unable to complete the baseline questionnaire within the first 4 days after patient admission
- If an enrolled participant is unable to complete the quality of care questionnaires within the first 90 days after the patients’ ICU discharge (end of time window for T1, see the “Timing of outcome assessments” section).
- If a declined patient general consent form for the use of clinical routine data for research is discovered after the family member has been enrolled into the trial, and the patient subsequently does not agree to the specific use of their routine clinical data for this trial, and
- If a patient’s actual ICU stay lasted less than the expected 48 h

We will report the number and percentage of participants or patients with these protocol deviations by treatment (intervention and control). Regarding the return of quality of care questionnaires, we will additionally report the number and percentage of participants who returned the questionnaires later than 14 days but before 90 days, since 14 days were the originally planned end of the time window for T1 in the study protocol (see the “Timing of outcome assessments” section).

None of the violations will lead to the exclusion of the family member participant from the trial. However, some of the protocol deviations have consequences for the data collection and lead to missing data: (1) if participants return the baseline questionnaire after 4 days, only the baseline demographic data (that are not subject to change over time) will be recorded; (2) if participants return the quality of care questionnaires after 90 days, their answers will not be considered; (3) if patients decline the use of their routine clinical data, no patient data will be extracted and used in the analysis.

### Analysis populations

We will adhere to the intention-to-treat principle at the cluster-level and participant-level as much as possible. Clusters and their participants will be analyzed by the treatment to which they were assigned. Should a patient be transferred to another cluster (other ICU), the family member participant will be analyzed in the original cluster. This will be possible due to individual informed consent. Our analysis population will thus consist of all participants who were included in the trial and who met the eligibility criteria. See the “Missing data” section for handling of missing data.

## Trial population

### Screening data

All family members who either (1) entered the study (2) were considered non-eligible, or (3) were eligible but not enrolled into the study, are documented in a screening log. Furthermore, the participation of each family member is documented in the enrollment log. At screening, sex and year of birth are collected for the patients and their family members as well as their reason for non-participation (eligibility criteria not met, not invited to participate, refused to participate, other reasons). We plan to report the number of screened patients and family members, with reasons for non-participation, because sex and year of birth do not provide sufficient information to evaluate the representativeness of the trial sample.

### Eligibility

#### Cluster-level eligibility criteria

Study ICUs need to be able to provide the highest level of patient care and treat patients who are hemodynamically unstable, require ventilation with multiple-organ failure, and need multidisciplinary intervention. They may offer different or combined specialty care, including surgical, trauma, medical, cardiac, or neurological care. ICUs certified by the Swiss Society for Intensive Medicine (SGI) to operate at least eight beds are eligible. ICUs with fewer than 300 admissions per year of patients with a length of stay of 48 h or more in the ICU are excluded, as are ICUs with a preexisting, protocolized, interprofessional family support program.

#### Participant-level eligibility criteria

Participants are adult family members ($\ge$ 18 years of age) of critically ill persons who are admitted to an eligible ICU with no preexisting declined general informed consent for use of their clinical data for research purposes. Patient-level inclusion criteria are having an expected length of stay in ICU of $\ge$ 48 h, as appraised by the intaking ICU clinician (physician or nurse), and (1) a life-threatening condition with a high risk of death or long-lasting functional impairment or (2) a high risk of prolonged mechanical ventilation (> 24 h) as appraised by the intaking ICU clinician. Patient-level exclusion criteria are a pre-existing declined general consent or an actual ICU stay < 48 h. Family members are defined as close others from the patient’s perspective, as noted in clinical records or advanced directives, or as legally defined surrogate decision-makers. Legal or blood kinship is not a requirement. They have to be a primary support person for the critically ill person, sign a written informed consent form, and be able to complete the baseline data collection and family-reported questionnaires in German within the required time frame. Family members of patients with refused general consent will not be invited to take part. Family members with prior inclusion in the FICUS trial in another ICU, with cognitive inability to understand the study or inability to complete the questionnaire as appraised by clinicians or study recruitment staff, will be excluded.

It should be noted that we do no longer plan to exclude family members of patients with an actual stay < 48 h on the study ICU after meeting the inclusion criteria of expected length of ICU stay of $\ge$ 48 h, even though this was originally defined as exclusion criterion. This is a protocol deviation defined in the “Adherence and protocol deviations” section.

### CONSORT flow diagram

**Fig. 1:**
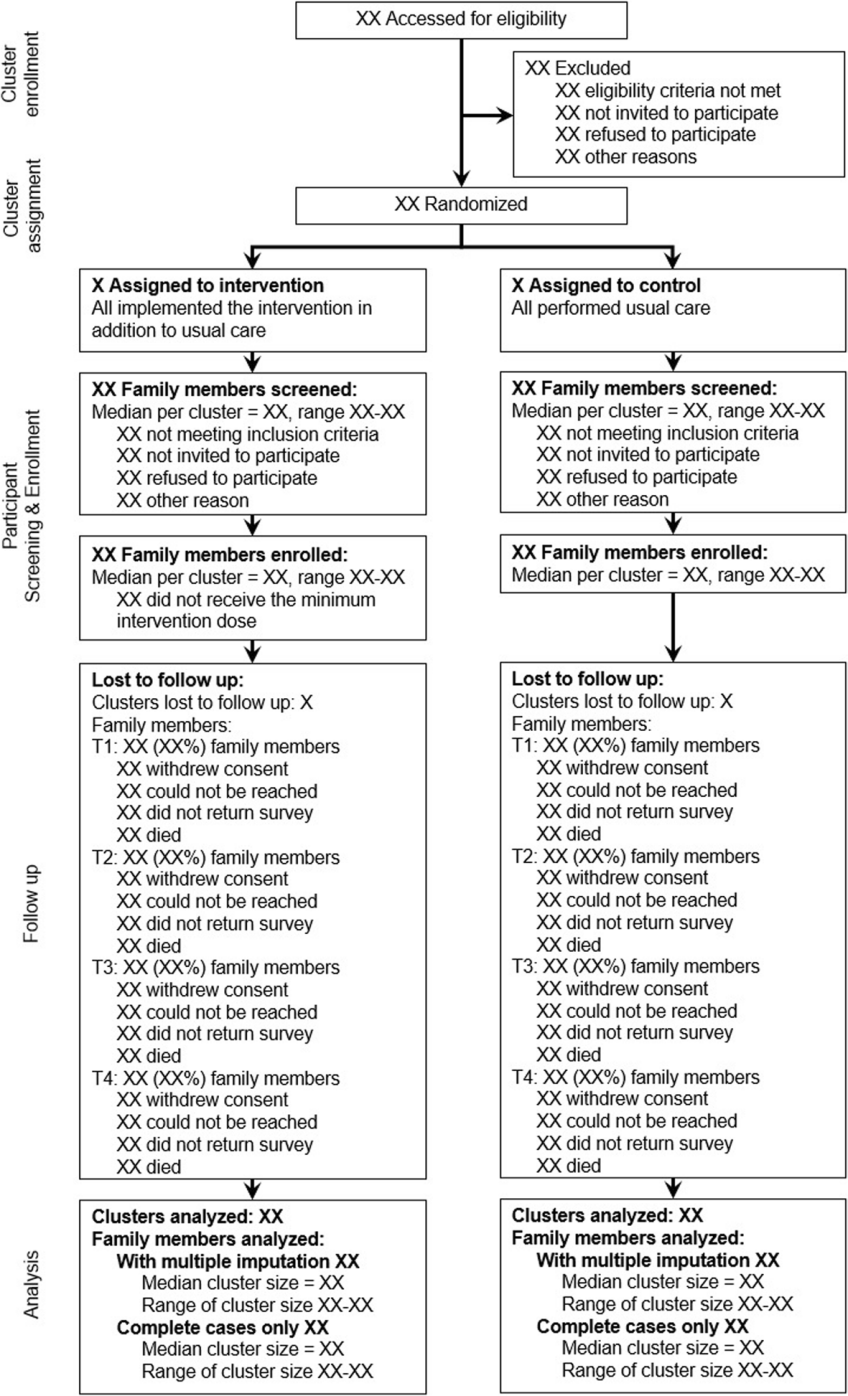
CONSORT flow diagram for the FICUS trial

Figure 1 shows the CONSORT flow diagram we plan to report for the FICUS trial.

### Withdrawal/follow-up

Withdrawal of consent by the study participants leads to the termination of their data collection. The data collected up to that point in time will be used in the analysis. If a patient withdraws consent for the use of clinical data for the study, no new clinical patient data will be collected. We will report the number and percentage of participants who withdraw informed consent and of patients who withdraw consent for their use of clinical data by study arm. In addition, we will report the number and percentage of participants who are lost to follow-up. We will further split these numbers by the timing of withdrawal/follow-up, e.g., before visit T1, T2, T3, or T4.

### Baseline characteristics

Baseline characteristics of the clusters (ICUs) before randomization will be summarized as outlined in Table 1 based on the most recent Minimal Data Set (MDSi) which includes data from the last calendar year before the start of the trial. Baseline characteristics of the patients and family member participants will be summarized as shown in Tables 2 and 3. Due to the relatively small number of clusters per arm, we will report medians together with minima and maxima for the cluster characteristics where appropriate (Table 1) but medians together with first and the third quartiles (q1 and q3) for the patient and family member characteristics (Tables 2 and 3).

**Table 1:** Baseline characteristics of clusters before randomization

Characteristic	Intervention arm *n *= 8	Control arm *n *= 8
Certification^a^ , *n* ( %)		
Major teaching hospitals (A/Au)	XX (XX %)	XX (XX %)
Other teaching hospitals (B)	XX (XX %)	XX (XX %)
Operated ICU beds (annual average), median (min, max)	XX (XX, XX)	XX (XX, XX)
Patients admitted, median (min, max)	XX (XX, XX)	XX (XX, XX)
Treatment days, median (min, max)	XX (XX, XX)	XX (XX, XX)
High-risk admissions (SAPS-2 score^b^ > 45), %, median (min, max)	XX (XX, XX)	XX (XX, XX)
Unplanned admissions, %, median (min, max)	XX (XX, XX)	XX (XX, XX)
SGI^c^ classification of treatment shifts, %, median (min, max)		
Category 1a	XX (XX, XX)	XX (XX, XX)
Category 1b	XX (XX, XX)	XX (XX, XX)
Category 2	XX (XX, XX)	XX (XX, XX)
Category 3	XX (XX, XX)	XX (XX, XX)
Primary diagnosis/treatment at admission, %, median (min, max)		
Cardiac	XX (XX, XX)	XX (XX, XX)
Respiratory	XX (XX, XX)	XX (XX, XX)
Gastrointestinal	XX (XX, XX)	XX (XX, XX)
Neurological	XX (XX, XX)	XX (XX, XX)
Metabolic-endocrine	XX (XX, XX)	XX (XX, XX)
Trauma	XX (XX, XX)	XX (XX, XX)
Urogenital	XX (XX, XX)	XX (XX, XX)
Other	XX (XX, XX)	XX (XX, XX)
Mechanically ventilated patients, %, median (min, max)	XX (XX, XX)	XX (XX, XX)
Mechanically ventilated patients > 95 h, %, median (min, max)	XX (XX, XX)	XX (XX, XX)
Patients with > 1000 NEMS^d^ points, %, median (min, max)	XX (XX, XX)	XX (XX, XX)
Length of stay (days), median (min, max)	XX (XX, XX)	XX (XX, XX)
Discharge destination, %, median (min, max)		
Other ICU	XX (XX, XX)	XX (XX, XX)
Intermediate care	XX (XX, XX)	XX (XX, XX)
General ward	XX (XX, XX)	XX (XX, XX)
Died	XX (XX, XX)	XX (XX, XX)
Other (rehabilitation or other care institution, home)	XX (XX, XX)	XX (XX, XX)
Nurse staffing (FTE/operated beds), %, median (min, max)	XX (XX, XX)	XX (XX, XX)
Staff with ICU certification, %, median (min, max)		
Nurses	XX (XX, XX)	XX (XX, XX)
Physicians	XX (XX, XX)	XX (XX, XX)
Family-centered care in ICU score^e^ , median (min, max)	XX (XX, XX)	XX (XX, XX)
Patient & family-centered care score^f^, median (min, max)	XX (XX, XX)	XX (XX, XX)

^a^Swiss Institute for Continuing Medical Education, classification see https://www.sgi-ssmi.ch/files/Dateiverwaltung/de/ressorts/quali/KDS%20Kommission%20Datensatz/SGI-Kat_20060309_d_2012.pdf

^b^Simplified Acute Physiology Score 2

^c^Swiss Society for Intensive Medicine

^d^Nine Equivalents of Nursing Manpower use Score

^e^Mean score (range 1–4, where 1 indicates the highest degree of family-centeredness) of 22 selected items from the gap analysis tool provided by the Society of Critical Care Medicine

^f^Mean score (range 1–5) of 30 selected items from the Patient- and Family-Centered Care Organizational Self-Assessment Tool

**Table 2:** Baseline characteristics of patients upon admission to ICU

Patient characteristic	Intervention arm *n *= XX	Control arm *n *= XX
Age (years), median (q1, q3)	XX (XX, XX)	XX (XX, XX)
Sex, n ( %)		
Female	XX (XX %)	XX (XX %)
Male	XX (XX %)	XX (XX %)
Other	XX (XX %)	XX (XX %)
Civil status, *n* ( %)		
Single	XX (XX %)	XX (XX %)
Married/in (registered) partnership	XX (XX %)	XX (XX %)
Divorced/separated	XX (XX %)	XX (XX %)
Widowed/surviving partner	XX (XX %)	XX (XX %)
Unplanned admission to ICU, *n* ( %)	XX (XX %)	XX (XX %)
Admitted from, *n* ( %)		
Emergency room	XX (XX %)	XX (XX %)
Operating room	XX (XX %)	XX (XX %)
General ward	XX (XX %)	XX (XX %)
Intermediate care	XX (XX %)	XX (XX %)
Other ICU	XX (XX %)	XX (XX %)
Other institution (rehabilitation, nursing home)	XX (XX %)	XX (XX %)
Mechanical ventilation, *n* ( %)	XX (XX %)	XX (XX %)
Mechanical circulatory support, *n* ( %)	XX (XX %)	XX (XX %)
SAPS-2^a^ score, median (q1, q3)	XX (XX, XX)	XX (XX, XX)
NEMS^b^ score, median (q1, q3)	XX (XX, XX)	XX (XX, XX)
SOFA^c^ score, median (q1, q3)	XX (XX, XX)	XX (XX, XX)
Trauma treatment, *n* ( %)	XX (XX %)	XX (XX %)
AIS^d^ score in case of trauma treatment, median (q1, q3)	XX (XX, XX)	XX (XX, XX)
Surgery, *n* ( %)		
Planned	XX (XX %)	XX (XX %)
Emergency	XX (XX %)	XX (XX %)
No surgery	XX (XX %)	XX (XX %)
Previous ICU-treatment within last 3 months, *n* ( %)	XX (XX %)	XX (XX %)

^a^Simplified Acute Physiology Score 2

^b^Nine Equivalents of Nursing Manpower use Score

^c^Sequential Organ Failure Assessment Score

^d^Abbreviated Injury Scale Score

**Table 3:** Baseline characteristics of family members participating in the study

Family member characteristic	Intervention arm *n* = XX	Control arm *n* = XX
Age (years), median (q1, q3)	XX (XX, XX)	XX (XX, XX)
Sex, *n* ( %)		
Female	XX (XX %)	XX (XX %)
Male	XX (XX %)	XX (XX %)
Other	XX (XX %)	XX (XX %)
Civil status, *n* ( %)		
Single	XX (XX %)	XX (XX %)
Married/in (registered) partnership	XX (XX %)	XX (XX %)
Divorced/separated	XX (XX %)	XX (XX %)
Widowed/surviving partner	XX (XX %)	XX (XX %)
Occupational status, *n* ( %)		
Employed (full- or part-time)	XX (XX %)	XX (XX %)
Retired	XX (XX %)	XX (XX %)
Student	XX (XX %)	XX (XX %)
Unemployed	XX (XX %)	XX (XX %)
Type of family member, *n* ( %)		
Partner/spouse	XX (XX %)	XX (XX %)
Parent	XX (XX %)	XX (XX %)
Child	XX (XX %)	XX (XX %)
Other	XX (XX %)	XX (XX %)
Co-habiting with patient, *n* ( %)	XX (XX %)	XX (XX %)
Travel time to hospital (minutes), median (q1, q3)	XX (XX, XX)	XX (XX, XX)
Self-perceived health (VAS^a^, 0-100), median (q1, q3)	XX (XX, XX)	XX (XX, XX)
Past/current psychiatric or psychological treatment, *n* ( %)	XX (XX %)	XX (XX %)
Current use of prescription drugs, *n* ( %)	XX (XX %)	XX (XX %)
Current treatment for chronic illness, *n* ( %)	XX (XX %)	XX (XX %)
Prior ICU experience, *n* ( %)		
As patient	XX (XX %)	XX (XX %)
As family member of patient	XX (XX %)	XX (XX %)
Both	XX (XX %)	XX (XX %)
None	XX (XX %)	XX (XX %)

^a^Visual analog scale

## Analysis

### Outcome definitions

The primary outcome is the quality of family care in the ICU, operationalized as family satisfaction with ICU care, which is an established core indicator of the quality of family care. It will be assessed at discharge from ICU by the Family Satisfaction in ICU Questionnaire (FS-ICU-24R) [27, 28].

Secondary outcomes are shown together with the primary outcome in Table 4 (adapted from [22], where more detail is given), which also provides the range of each outcome, Cronbach’s $\alpha$ (where applicable), and the timing of each assessment.

**Table 4:** Primary and secondary outcomes measures

Domain/construct	Measure^a^	Range	$\alpha$^b^	T0	T1	T2–T4
Quality of family care						
Satisfaction with care (primary outcome)	Family satisfaction with ICU Care (FS-ICU-24R)	0–100	0.96^c^		X	
Subscales of FS-ICU-24R	FS-ICU-24R Care (subscale) score	0–100	0.95^d^		X	
	FS-ICU-24R Decision-Making (subscale) score	0–100	0.87^d^		X	
Quality of communication	Questionnaire on Quality of Physician–Patient Interaction (QQPPI-14)	1–5	0.95		X	
Support from nurses	Family Perceived Support Questionnaire (ICE-FPSQ-14)	14–70	0.92^e^		X	
Family management						
Family functioning	Family Assessment Device - General Functioning Scale (FAD-GF-12)	1–4	0.87	X	X	X
Family resilience	Brief Resilience Scale (BRS-6)^f^	1–5	0.85	X	X	X
Mental health						
Subjective well-being	Satisfaction with Life Scale (SWLS-5)	5–35	0.89–0.92	X	X	X
	WHO-5 Well-Being Index (WHO-5)	0–100	0.92	X	X	X
	Adapted VAS on Quality of Life (QoL-VAS)	0–100	n/a	X	X	X
Psychological distress	Distress Thermometer (DT)	0–10	n/a	X	X	X
	Impact of Events Scale-6 (IES-6)	0–4	0.80	X	X	X
	Hospital Anxiety and Depression Scale (HADS-14)	0–21	> 0.80	X	X	X

^a^German versions of measures

^b^Cronbach’s $\alpha$, most references are listed in the study protocol [22], except for some updates

^c^Cronbach’s $\alpha$ according to [27], for the English version

^d^Cronbach’s $\alpha$ according to [28]

^e^Cronbach’s $\alpha$ according to [29]

### Analysis methods

#### Primary outcome―main analysis

The FS-ICU-24-R total score at patient discharge from ICU will be analyzed by a linear mixed-effects model (LMM) with a random intercept per cluster to account for the non-independence of family members from the same cluster. Due to the small number of clusters, the main model (model 1) will include the treatment (intervention vs. control) as the only explanatory variable, and the Satterthwaite approximation for the denominator degrees of freedom will be used, as was recommended [30] for cluster-randomized trials with 10–20 clusters randomized. The ICC will be estimated from this model based on the residual variance and between-cluster variance.

#### Primary outcome―sensitivity analyses

The following covariate-adjusted sensitivity analyses will be conducted to adjust the treatment effect estimate for potential confounding: At the cluster level, the ICU certification (as used in the cluster randomization), overall ICU nurse staffing (ratio of nurses full-time equivalents to the number of certified ICU beds), and the family-centered care in ICU score (mean score of 22 items from the Standardized score sheet provided by the Society of Critical Care Medicine [31], ranging from 1 to 4) will each be added separately to the main model described above (models 2–4). The hospital, also used in the cluster randomization, will not be accounted for, neither as a covariate nor as a random term, because most ICUs are in a different hospital (see the “Randomization” section).

At the individual participant level, patient age, cause of admission (unplanned or planned), the SAPS-2 score of the patient (mortality risk), type of relationship between patient and family member (partner, child or other), and the family member’s previous ICU experience will be added together to the main model described above (model 5) and to the cluster-level adjusted models (models 6–8).

To assess the sensitivity of the results with regard to missing data (see also the “Missing data” section), we will apply model 1 and model 5 as described above to a multiply imputed data set (models 9 and 10). We may further apply models 6–8 to the multiply imputed data set.

#### Primary outcome―subgroup analyses

Subgroup analyses are planned regarding the primary outcome for the following baseline characteristics:

Cluster characteristics

- Certification (as defined in the “Randomization” section)
- Overall ICU nurse staffing
- Family-centered care in ICU score (mean score, range 1–4)

Patient or family member characteristics

- Patient age
- Patient sex
- Patient’s cause of admission (unplanned vs. planned)
- Mortality risk of patient, as assessed by the SAPS-2 score
- Type of relationship between patient and family member (partner, child, other)
- Family member’s prior ICU experience (yes/no)
- Family resilience, as assessed by the Brief Resilience Scale (BRS-6)
- Family functioning, as assessed by the Family Assessment Device - General Functioning Scale (FAD-GF-12)
- Family member’s anxiety score assessed by the Hospital Anxiety and Depression Scale (HADS)
- Family member’s depression score assessed by HADS

Subgroup effects will be tested by a separate LMM fitted to the primary outcome for each subgroup variable, adding the subgroup variable and its interaction with the treatment as explanatory variables to the main model described above. A significant interaction would indicate a different treatment effect depending on the subgroup (or along a gradient for the continuous subgroup variables).

#### Primary outcome―additional analyses

To investigate the effect of intervention fidelity, three models that each include a certain type of intervention fidelity will be fitted to the primary outcome. The explanatory variable treatment (intervention vs. control) in model 5 will be replaced by one of the following explanatory variables:

- Consistency of intervention delivery with three levels: consistent delivery in the intervention arm, inconsistent delivery in the intervention arm and usual care in the control arm. Intervention delivery is defined as consistent if the minimal intervention contact dose according to protocol was provided (see the “Adherence and protocol deviations” section)
- Volume (total duration of interventions (conversations) divided by patient length of stay at ICU), which is zero in the control arm
- Frequency (total number of interventions (conversations) divided by patient length of stay at ICU), which is zero in the control arm

These models will also be applied to the multiply imputed data set.

#### Secondary outcomes

The secondary outcomes regarding the quality of care, which are only measured once at discharge from ICU (T1), will be analyzed with an LMM as described above for the primary outcome. All other secondary outcomes, which are measured at baseline (T0) and four times after the start of the intervention (T1–T4), will be analyzed by an LMM with a random intercept per cluster and a random intercept per family member (nested within clusters) to additionally account for the non-independence of repeated measurements from the same study participant. The serial autocorrelation of residuals will be modeled using a first-order autoregressive correlation structure. The models will include the treatment (intervention vs. control), the corresponding baseline measurement, the visit, and the visit $\times$ treatment interaction to assess whether the treatment effect changes over time.

Depending on the results of the sensitivity analyses for the primary outcome, we will use the same covariate-adjusted sensitivity analyses (or some of them) also for the secondary outcomes.

#### Presentation of outcome data and effect size estimates

Outcome data will be presented in a table containing descriptive statistics of each outcome per trial arm as well as point estimates and 95% confidence intervals for treatment effect estimates and corresponding *p*-values.

### Missing data

We will analyze complete cases in the main analysis and use multilevel multiple imputation of missing data (outcomes and covariates) at the participant level ([32], Chapter7) in sensitivity analyses (the “Analysis methods” section). We do not expect any missing cluster level covariates. We will separately impute missing values in the control and intervention arm, as recommended by [33]. The dataset used for multiple imputation will be a subset of the full data set, containing (1) the primary outcome, (2) all cluster and participant level covariates that are used in the planned analyses of the primary outcome (see the “Analysis methods” section), (3) secondary quality of care outcomes (only measured at T1) due to an expected correlation with primary outcome, and (4) once known which covariates contain missing data, additional variables that are correlated with the missing covariate data. The number of imputations per missing value will be determined based on the fraction of missing information [34]. We do not expect any clusters to be missing as a whole, but would exclude them from the analysis if present.

### Additional analyses

Subgroup analyses as defined for the primary outcome (see the “Analysis methods” section) will be performed for each of these secondary outcomes:

- Quality of communication Questionnaire on Quality of Physician-Patient Interaction (QQPPI-14), T1
- Impact of Events Scale-6 (IES-6), T1-T4
- Hospital Anxiety and Depression Scale (HADS-14): Anxiety Subscale, T1-T4
- Hospital Anxiety and Depression Scale (HADS-14): Depression Subscale, T1-T4

Additional analyses, similar to those defined for the primary outcome (see the “Analysis methods” section), will be performed for all secondary outcomes.

Due to the repeated measurements for some of these secondary outcomes, the corresponding models will additionally include a random intercept per family member and the corresponding baseline measurement per variable as covariate (as described in the “Analysis methods” section) but will not include the visit and the visit $\times$ treatment interaction. Should the analyses of secondary outcomes (the “Analysis methods” section) reveal that an intervention effect only manifests at certain follow-up times, we would focus the described additional analyses of secondary outcomes on these follow-up times.

In addition, we will compute Cronbach’s alpha for total scores and subscales of all family-reported outcome measures used in our study.

### Harms

The intervention applied in this trial cannot cause any harm to the patient, as it is applied to the family members of the patient and is a family care intervention, not a medical intervention. However, we will report all SAEs, for example if a study participant (family member) dies during the follow-up period and is thus lost to follow-up.

### Statistical software

All statistical analyses will be performed using the R system for statistical computing and graphics [24] (current version at the time of analysis). The linear mixed-effects models will be fitted using the lmer function from the R package lme4 [35] in combination with the package lmertest which implements the Satterthwaite method for the degrees of freedom [36]. Multiple imputation will be performed using the R package mice [37] in combination with the 2l.pmm method from the miceadds package for the multilevel imputation [38]. All software used will be reported together with the results of the analyses.

## Data Availability

Data sharing is not applicable to this article as no datasets were generated or analyzed during the current study (statistical analysis plan).
